# Congenital Epidermal Nevus

**DOI:** 10.5005/jp-journals-10005-1232

**Published:** 2014-04-26

**Authors:** Bhawna Arora, Vineet Inder Singh Khinda, Nitika Bajaj, Gurlal Singh Brar

**Affiliations:** Senior Lecturer, Department of Pediatric Dentistry, Adesh Institute of Dental Sciences and Research, Bathinda, Punjab, India; Professor and Head, Department of Pediatric Dentistry, Genesis Institute of Dental Sciences and Research, Ferozepur, Punjab, India; Reader, Department of Pediatric Dentistry, Genesis Institute of Dental Sciences and Research, Ferozepur, Punjab, India; Senior Lecturer, Department of Pediatric Dentistry, Genesis Institute of Dental Sciences and Research, Ferozepur, Punjab, India

**Keywords:** Epidermal, Nevus, Nevi, Cryosurgery, Dermabrasion

## Abstract

Epidermal nevi are hamartomas that are characterized by hyperplasia of the epidermis and adnexal structures, and may be associated with serious disfiguration. Germline mutations in the FGFR3 gene have found to be the etiology of epidermal nevus. Patients often seek treatment from dermatologic surgeons but even an alert dentist can help to diagnose the lesion from its clinical appearance. Various treatment modalities are available and it is the clinician's choice to choose depending upon the patient's condition.

**How to cite this article: **Arora B, Khinda VIS, Bajaj N, Brar GS. Congenital Epidermal Nevus. Int J Clin Pediatr Dent 2014;7(1): 43-46.

## INTRODUCTION

An epidermal nevus (plural: nevi) is an abnormal, non-cancerous (benign) patch of skin caused by an overgrowth of skin cells. It is typically seen at birth or develop in early childhood and evolve until puberty.^1^ These are hamartomas that are characterized by hyperplasia of the epidermis and adnexal structures and may be associated with serious disfiguration.^2^ They can be divided into either nonorganoid (keratinocytic) types or organoid types characterized by hyperplasia of adnexal structures, such as sebaceous glands, sweat glands and hair follicles. Epidermal nevi of the common, nonorganoid and nonepidermolytic type are benign skin lesions and may vary in their extent from a single (usually linear) lesion to widespread and systematized involvement. The subtypes of this tumor differ according to the distribution of the lesions or the predominant histologic cell type: keratinocyte (verrucous epidermal nevus), sebaceous gland (nevus sebaceous), pilosebaceous unit (nevus comedonicus), eccrine gland (eccrine nevus), or apocrine gland (apocrine nevus).^3,4^ The most common type of epidermal nevi is the keratinocytic nevi, also called verrucous epidermal nevi. They are usually present at birth or infancy and may enlarge slowly during childhood. The nevus typically appears as verrucous papules that coalesce to form well-demarcated, skin-colored, brownish or gray-brown, papillomatous plaques.^5,6^

### Prevalence, Epidemiology and Etiology

Epidermal nevi show a prevalence of about one in 1,000 live births and affect males and females equally.^1,3,5^ An estimated one-third of individuals with epidermal nevi have involvement of other organ systems; hence, this condition is considered to be an epidermal nevus syndrome (ENS) and it has been reported that up to 10% of individuals with epidermal nevi may develop additional syndrome symptoms. This syndrome is usually apparent at birth (due to the skin lesions which are most often seen in the midface from the forehead down into the nasal area) and is often associated with seizures, mental deficiency, eye problems, bone mal­formations and atrophy of the brain.^7^

These lesions usually result from a postzygotic mutation in an embryonic cell that is destined to populate an area of the epidermis and exhibit Blaschko's lines.^1,2^ Mutations in the fibroblast growth factor receptor 3 (FGFR3) gene have been found in approximately 30% of people with a type of nevus in the keratinocytic epidermal nevi group. The gene mutations involved in most epidermal nevi are unknown. Mutations associated with an epidermal nevus are present only in the cells of the nevus, not in the normal skin cells surrounding it. Because, the mutation is found in some of the body's cells but not in others, people with an epidermal nevus are said to be mosaic for the mutation. The FGFR3 gene provides instructions for the FGFR3 protein. This protein is involved in several important cellular processes, including regulation of growth and division of skin cells. The FGFR3 protein interacts with specific growth factors outside the cell to receive signals that control growth and development. When these growth factors attach to the FGFR3 protein, the protein is turned on (activated) which triggers a cascade of chemical reactions inside the cell that control growth and other cellular functions. The most common FGFR3 gene mutation in epidermal nevi creates a protein that is turned on without attachment of a growth factor, which means that the FGFR3 protein is constantly active. Cells with a mutated FGFR3 gene grow and divide more than normal cells. In addition, these mutated cells do not undergo a form of self-destruction called apoptosis as readily as normal cells. These effects result in overgrowth of skin cells, leading to epidermal nevi.^1,8-10^

## CLINICAL PRESENTATION

Epidermal nevi present as yellowish-brown, velvety, granu­lar or warty plaques. They may occur as single or multiple lesions and typically have a linear or whorled configuration, following the lines of Blaschko. Blaschko's lines are believed to represent the lines of migration of embryonal cells proliferating from the neural crest and are also adopted by mutated cells resulting in the linear appearance of the lesions.^1,11-13^ Nevi may be unilateral, bilateral or distributed on most of the body. It may arise anywhere on the cutaneous surface and may also involve the oral mucosa and ocular conjunctiva, which is rarely reported. Most epidermal nevi measure several centimeters or less in length but can extend along an entire limb or traverse the chest, abdomen or back. Malignant degeneration (e.g. basal cell carcinoma or squamous cell carcinoma) of epidermal nevi is rare.^7,12,13-15^

## CASE REPORT

**Figs 1A and B:**
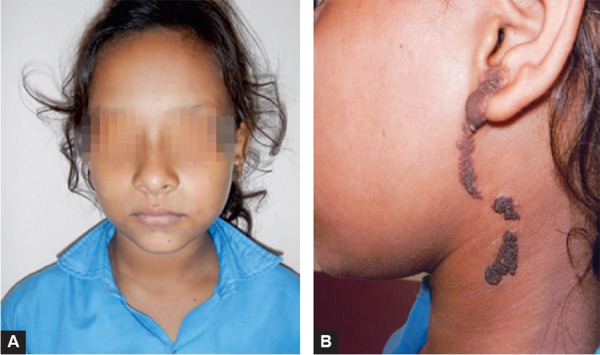
(A) Facial Profile of the patient and (B) extension of the lesion

A 10-year-old girl presented to the department of pediatric Dentistry for the routine dental examination. The oral and dental health was normal but there was a lesion on the left side of the skin of face. The lesion was present since birth. The lesion involved the left cheek region; near the preauricular region, extending from the ear lobe superiorly to cervical region inferiorly (Figs 1A and B). The lesion measured 6 × 1.5 cm in size. It was black in color, had a rough and warty surface and was nontender. The underlying skin appeared normal. It was nonpulsatile with no evidence of impulse on coughing. The regional lymph nodes were nonpalpable. The general examination of the body showed no such lesion on any other body part. There was no relevant family history.

The patient was referred to the department of oral surgery and otolaryngology for further assessment and biopsy. Routine blood investigations showed that her hemoglobin level was low and other counts, like BT and CT, were in normal range. A provisional diagnosis of some papillomatous viral infection or nevi was made. An incisional biopsy was performed under local anesthesia and histopathological examination of the swelling was done to establish a diagnosis.

### Histopathological report

**Fig. 2:**
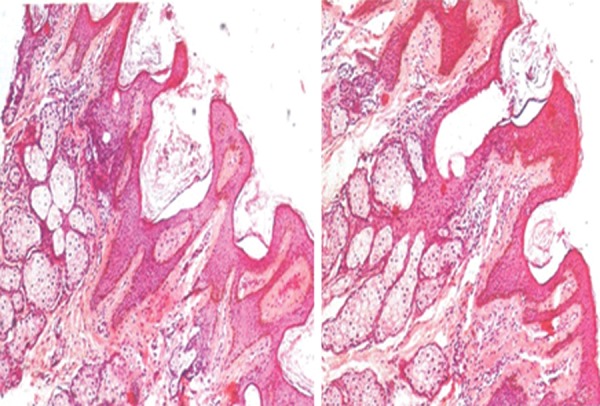
Histopathological examination at 4× and 10×

The histopathological examination (Fig. 2) exhibited epi­dermal acanthosis, mild papillomatosis, laminated hyper-keratosis and increased pigmentation of basal keratinocytes. Theques and clusters of nevus cells were seen. There was perinuclear vacuolization with irregular cellular boundaries and an increased number of irregular shaped large kera-tohyalin granules in stratum corneum which clearly pointed toward the diagnosis of epidermal nevus.

After the final diagnosis of the lesion, i.e. epidermal nevus, the patient was referred to the dermatologist and plastic surgeon for the treatment of the lesion.

## DISCUSSION

Epidermal nevus is a linear, persistent, pruritic plaque, usually first noted on a limb in early childhood. Originally described by Unna in 1896, a few patients were reported prior to 1971 when Altman and Mehregan delineated infammatory linear verrucous epidermal nevus as a distinct entity in 25 patients. They coined the name infammatory linear verrucous epidermal nevus, labeling it a clinical and histopathologic type of linear verrucous nevus that is often infammatory or psoriasiform. Infammatory linear verrucous epidermal nevus accounts for approximately 5% of patients with epidermal nevi.

Six different syndromes with epidermal nevi as part of them have been delineated. These include (1) proteus, (2) CHILD syndrome (congenital hemidysplasia with ichthy-osiform nevus and limb defect), (3) phakomatosis pigmen-tokeratotica, (4) sebaceous nevus, (5) Becker's nevus, and (6) nevus comedonicus syndromes.^13,14-18^

Happle^7^ has reported the typical linear configuration also on the head and neck area and found that oral mucosa involvement is extremely rare. Historically, since the first review of Brown and Gorlin who reported 24 patients with oral and cutaneous LEN, only 11 patients with oral involvement have been described in the medical literature and, among these, only three cases were reported with exclusive oral localization.

The treatment of epidermal nevi is challenging. Multiple medical and surgical treatments have been attempted, but no ideal or universally acceptable treatment has emerged. Corticosteroids applied under occlusion or by injection as well as Tretinoin (syn. vit A) cream applied topically, may sometimes be partially effective. A class of medicine called oral retinoids may be beneficial for the treatment of wide­spread epidermal nevi, but may require life-long therapy. The treatment of choice for small epidermal nevi is surgical excision. Superficial means of removal frequently result in recurrence. Aggressive approaches may be more successful, but also carry a higher risk of postoperative scarring. Surgical excision, dermabrasion, cryosurgery, electrosurgery and laser surgery have each been used to treat epidermal nevi. Surgical excision always result in scar formation and thus is reserved for the small-sized lesions.^6,19-21^ Dermabrasion, if superficial, is associated with a high rate of recurrence, and deep dermabrasion can result in thickened scars. Cryo-surgery has similar limitations with the risks, including slow healing, infection, swelling and not uncommonly, abnormal coloration of the skin. Physicians have been performing laser treatment on epidermal nevi for decades. Recent advances in laser technology have increased the ease, precision and safety of such treatments. Several reliable and effective methods for treating epidermal nevi with CO_2_, long pulsed Nd:YAG and 585 nm pulsed dye lasers have been developed. However, recurrences can occur months or years after removal of epidermal nevi by any method.^22-27^

## CONCLUSION

Patients with epidermal nevus often seek treatment from dermatologic surgeons but even an alert dentist can help to diagnose the lesion from its clinical appearance. After a multidisciplinary examination to rule out neurologic, ocular, skeletal and cardiovascular complications of epidermal syndrome, it is incumbent upon the physician to offer the most prudent and effective therapy for the cutaneous manifestations about which the patient initially sought treatment.
